# Supplementary material for: Convergent evolution through independent rearrangements in the primate amylase locus
Source: bioRxiv. 2025 Aug 15:2025.08.14.670395. Preprint. [Version 1] doi: 10.1101/2025.08.14.670395 (PMC12363942; doi:10.1101/2025.08.14.670395)
Supplement: 1 [file NIHPP2025.08.14.670395V1-supplement-1.pdf]

bioRxiv preprint doi: https://doi.org/10.1101/2025.08.14.670395; this version posted August 15, 2025. The copyright holder for this preprint (which was not certified by peer review) is the author/funder, who has granted bioRxiv a license to display the preprint in perpetuity. It is made available under aCC-BY 4.0 International license.

Supplementary Materials for

Convergent evolution through independent rearrangements in the primate amylase locus

Charikleia Karageorgiou *et al.*

*Corresponding author. Email: Omer Gokcumen, omergokc@buffalo.edu

This PDF file includes:

Figs. S1 to S11
Tables S1 to S14 (Table titles only)

Other Supplementary Materials for this manuscript include the following:

Tables S1 to S14 (full data) provided as a separate Excel file

bioRxiv preprint doi: https://doi.org/10.1101/2025.08.14.670395; this version posted August 15, 2025. The copyright holder for this preprint (which was not certified by peer review) is the author/funder, who has granted bioRxiv a license to display the preprint in perpetuity. It is made available under aCC-BY 4.0 International license.

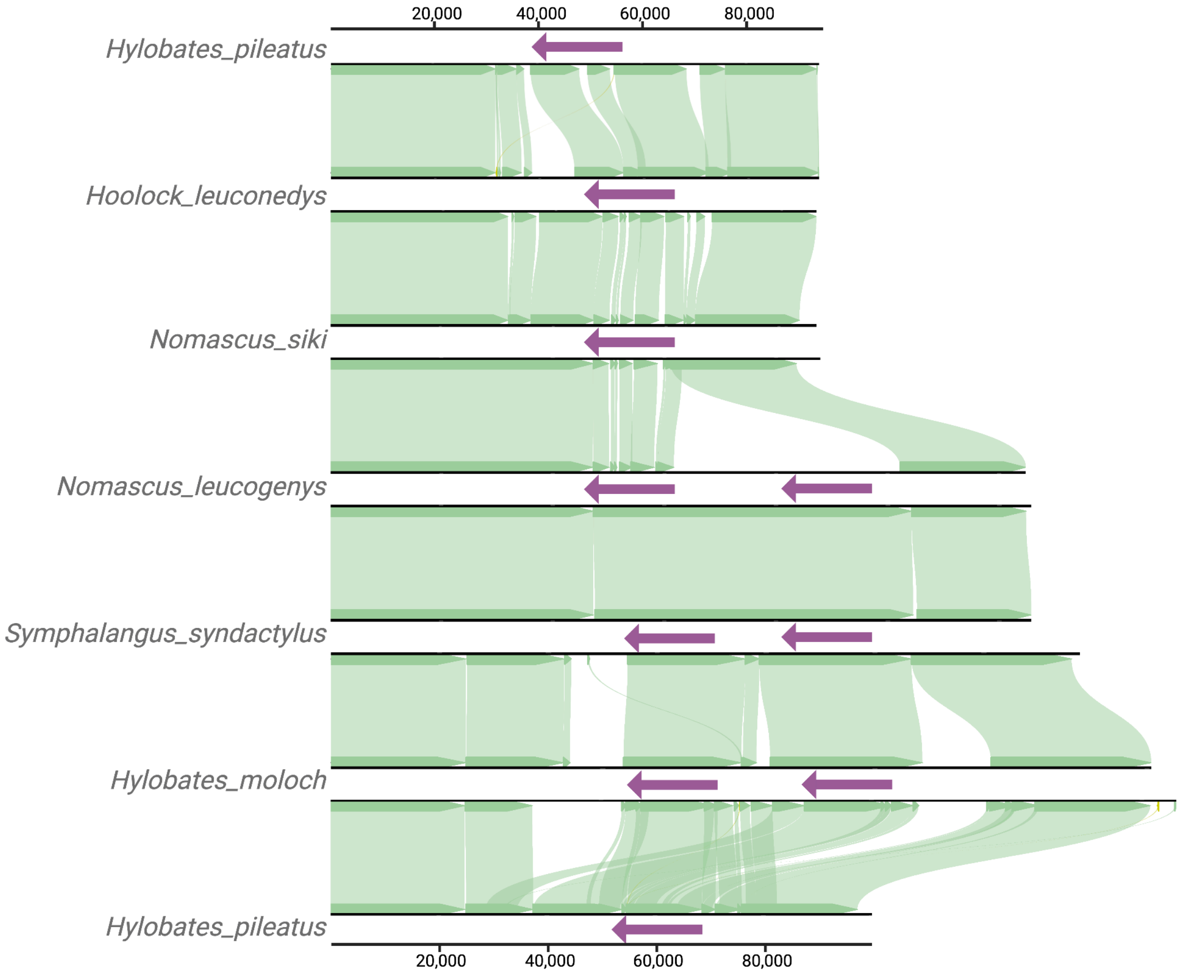

Fig. S1.

Copy-number and structural diversity of the amylase locus in gibbons (lesser apes). Miropeat style plots across representatives of *Hoolock leucodenys*, *Symphalangus syndactylus*, *Hylobates pileatus,* *Hylobates moloch* and *Nomascus leucogenys* and *Nomascus siki* illustrate lineage-specific differences consistent with either independent losses or incomplete lineage sorting (see main text). Axes denote locus coordinates.

bioRxiv preprint doi: https://doi.org/10.1101/2025.08.14.670395; this version posted August 15, 2025. The copyright holder for this preprint (which was not certified by peer review) is the author/funder, who has granted bioRxiv a license to display the preprint in perpetuity. It is made available under aCC-BY 4.0 International license.

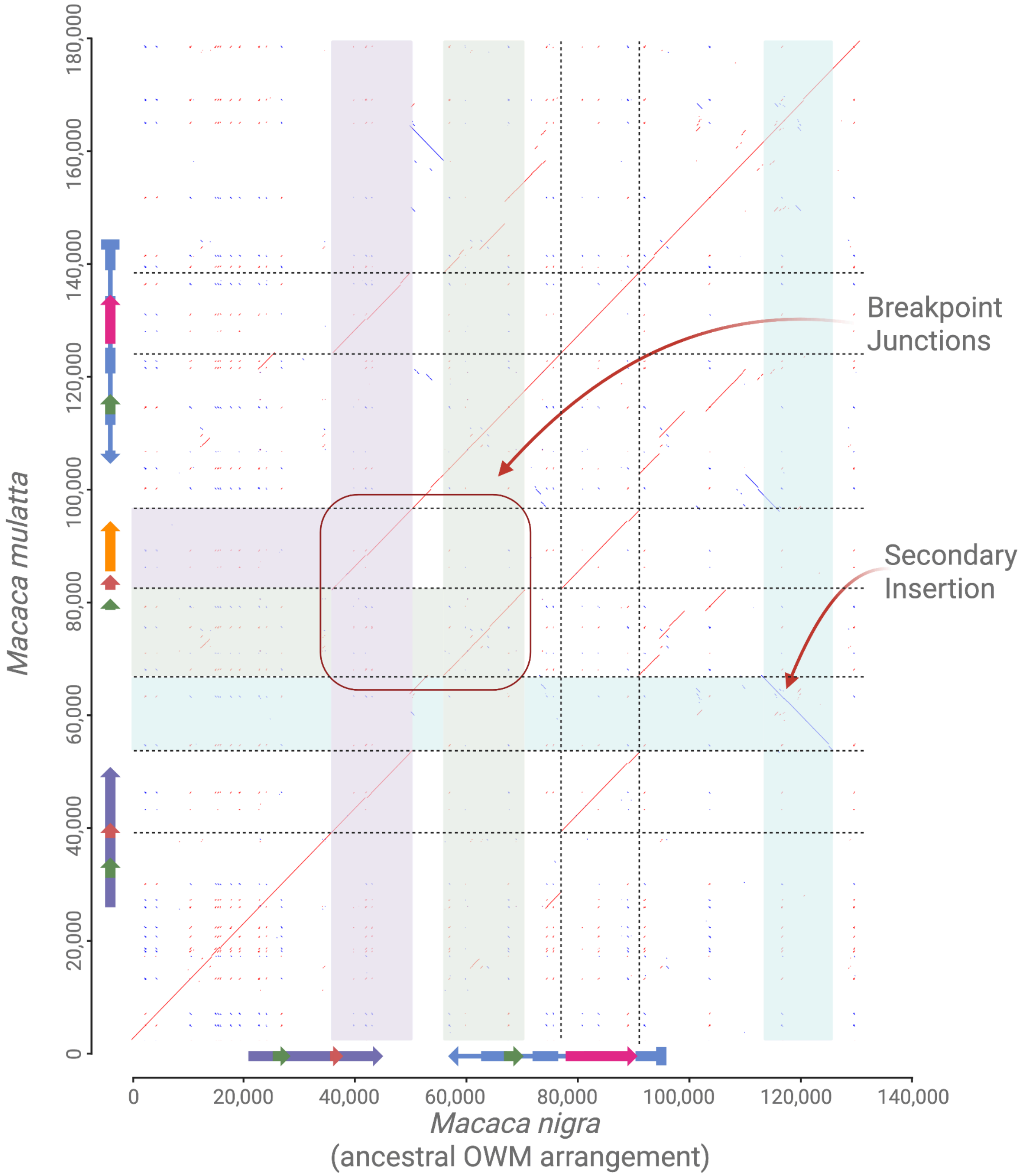

Fig. S2.

Synteny of the macaque amylase locus across *Macaca* and mapping of the *AMYm* duplication. Comparative dotplots use *Macaca nigra* as the two-copy ancestral Old World monkey configuration and highlight the novel *AMYm* block in the fascicularis and sinica clades (represented by *M. mulatta*), with breakpoints marked at the NAHR junction and a small secondary insertion within the novel duplicon.

bioRxiv preprint doi: https://doi.org/10.1101/2025.08.14.670395; this version posted August 15, 2025. The copyright holder for this preprint (which was not certified by peer review) is the author/funder, who has granted bioRxiv a license to display the preprint in perpetuity. It is made available under aCC-BY 4.0 International license.

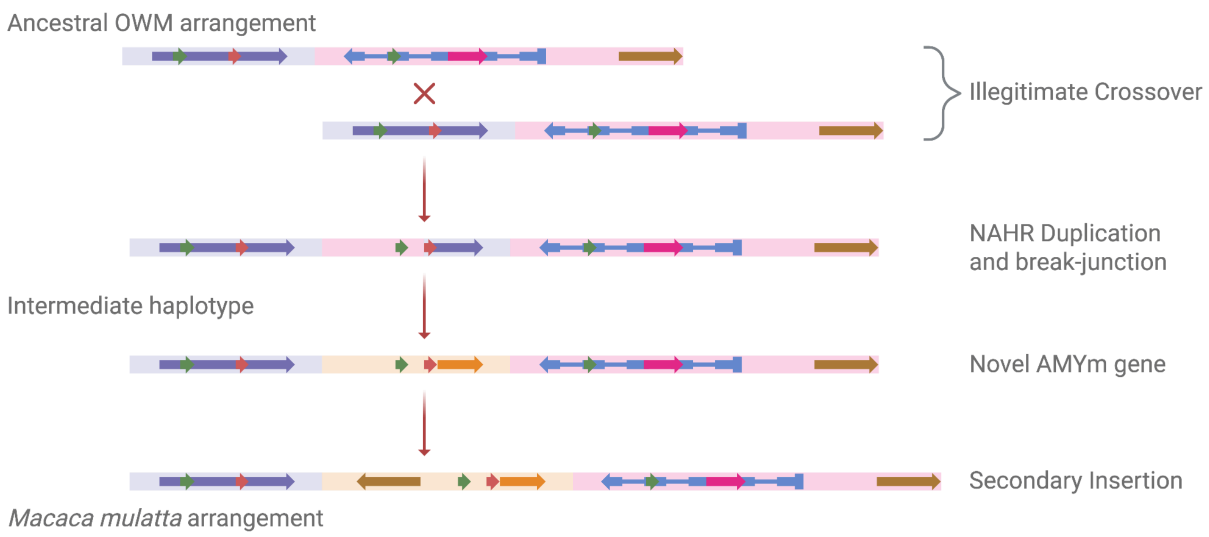

Fig. S3.

Mechanistic model for the macaque *AMYm* origin. Cartoon summarizing an initial illegitimate crossover (NAHR) between *AMY1′* (5′ homology) and *AMY2B* (3′ homology), generating the chimeric *AMYm* (intermediate haplotype). Following the NAHR duplication the locus experienced a small secondary insertion upstream the *AMYm*. Panels show the ancestral OWM configuration, *Macaca mulatta* arrangement, and the inferred intermediate arrangement prior to the secondary insertion.

bioRxiv preprint doi: https://doi.org/10.1101/2025.08.14.670395; this version posted August 15, 2025. The copyright holder for this preprint (which was not certified by peer review) is the author/funder, who has granted bioRxiv a license to display the preprint in perpetuity. It is made available under aCC-BY 4.0 International license.

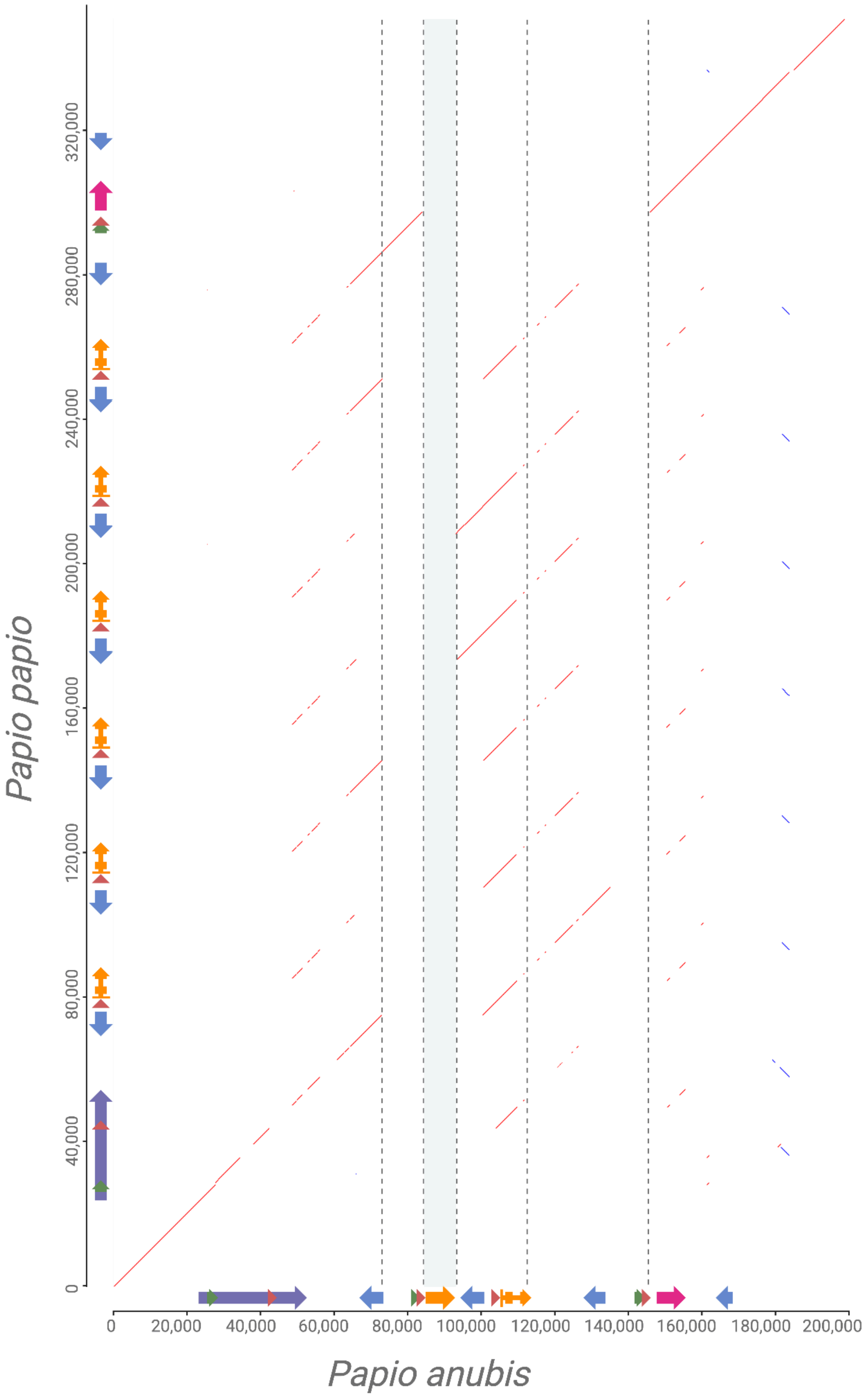

Fig. S4.

Dotplot of the amylase locus between *Papio anubis* (olive baboon; x-axis) and *Papio papio* (Guinea baboon; y-axis). *P. papio* lacks the *AMYp2* segment present in *P. anubis*, confirming *AMYp1* as the first and older duplication shared by both species. In *P. papio*, multiple tandem

bioRxiv preprint doi: https://doi.org/10.1101/2025.08.14.670395; this version posted August 15, 2025. The copyright holder for this preprint (which was not certified by peer review) is the author/funder, who has granted bioRxiv a license to display the preprint in perpetuity. It is made available under aCC-BY 4.0 International license.

tracts align identically to *AMYp1* (6 copies), a pattern consistent with assembly over-expansion rather than a true biological increase (see Methods). Alignments were generated with LAST (using lastal) and filtered with last-split to retain only primary, best-scoring placements and the dotplot was rendered from these primary chains. Breakpoint-proximal, single-copy flanks were used as anchors, increasing confidence that the observed absence of *AMYp2* in *P. papio* reflects genuine synteny rather than paralogous cross-mapping.

bioRxiv preprint doi: https://doi.org/10.1101/2025.08.14.670395; this version posted August 15, 2025. The copyright holder for this preprint (which was not certified by peer review) is the author/funder, who has granted bioRxiv a license to display the preprint in perpetuity. It is made available under aCC-BY 4.0 International license.

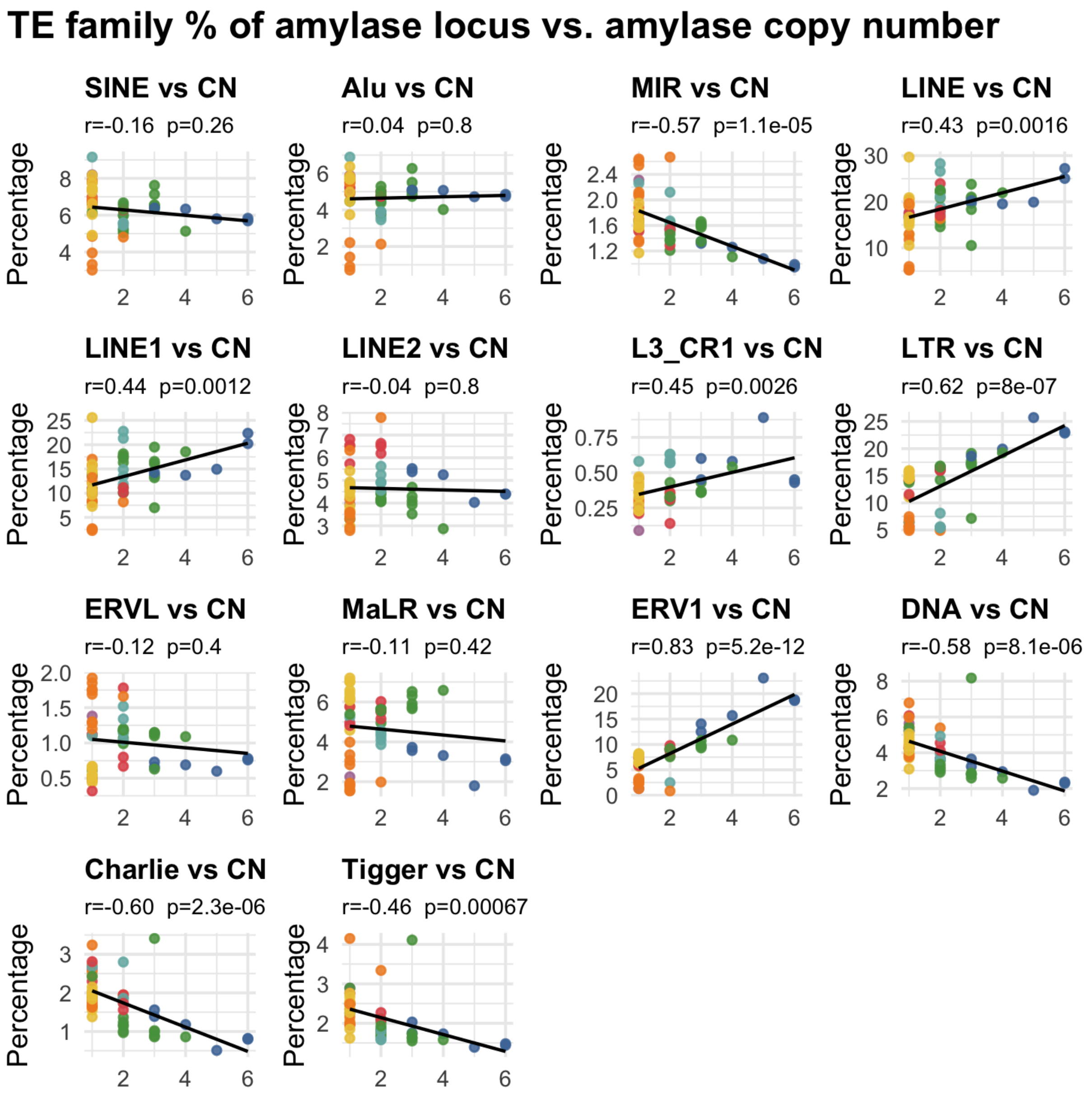

Fig. S5.

TE family abundance in the amylase locus versus AMY copy number across primates. Scatterplots show, for 53 species, the percentage of each TE family within the RNPC3-AMY1C amylase interval (y-axis) against the total AMY copy number per haploid genome (x-axis). Points are species (colored by clade as in Fig. 1); lines are least-squares fits. Panels report Pearson's r and two-sided P test. LTRs, LINEs (LINE1, L3/CR1) and ERV1 show positive correlations with AMY copy number, whereas MIRs and DNA transposons (Charlie, Tigger) are negatively correlated. TE content was called with RepeatMasker using a uniform primate library on locus-bounded sequences.

bioRxiv preprint doi: https://doi.org/10.1101/2025.08.14.670395; this version posted August 15, 2025. The copyright holder for this preprint (which was not certified by peer review) is the author/funder, who has granted bioRxiv a license to display the preprint in perpetuity. It is made available under aCC-BY 4.0 International license.

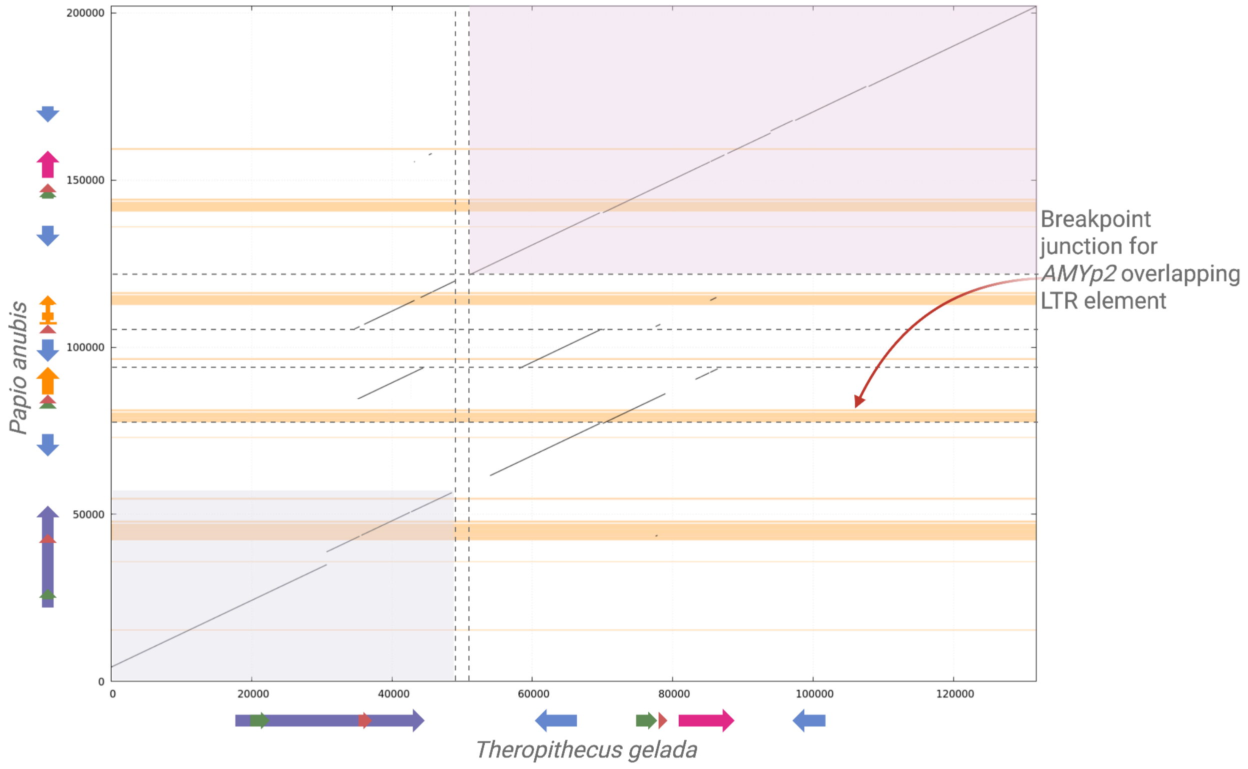

Fig. S6.

Dotplot of the olive baboon (*Papio anubis*) amylase locus (y-axis) versus the inferred ancestral Old World monkey two-copy locus from *Theropithecus gelada* (x-axis), aligned with LAST (primary alignments only). Light purple background blocks mark the two ancestral OWM segments. Horizontal orange bands are LTR annotations in olive baboons. The dashed horizontal lines mark the inferred crossover positions. The labeled *AMYp2* breakpoint junction overlaps an LTR tract (LTR25-int), consistent with LTR-seeded homology at the recombination site.

bioRxiv preprint doi: https://doi.org/10.1101/2025.08.14.670395; this version posted August 15, 2025. The copyright holder for this preprint (which was not certified by peer review) is the author/funder, who has granted bioRxiv a license to display the preprint in perpetuity. It is made available under aCC-BY 4.0 International license.

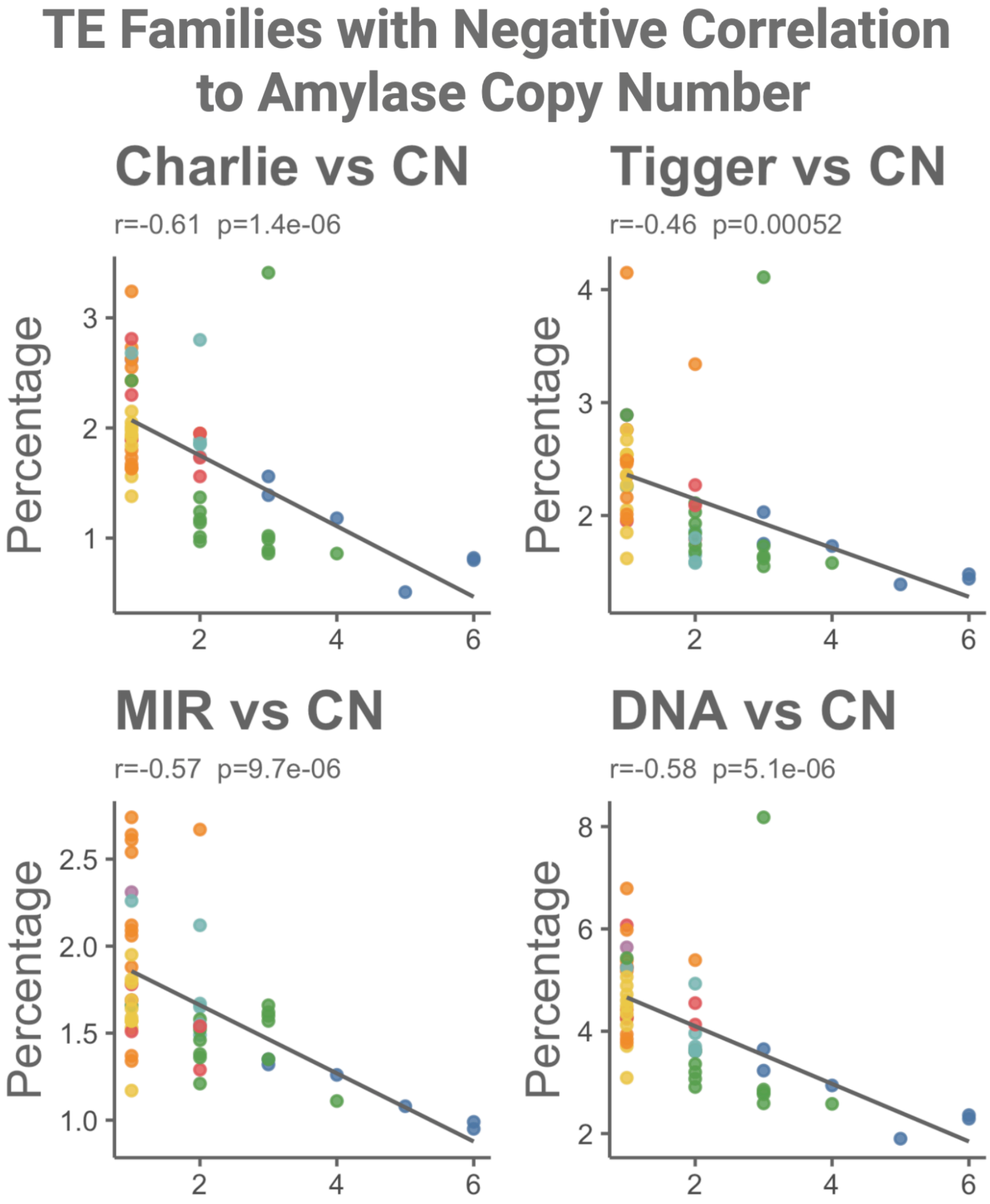

Fig. S7.
TE families negatively correlated with AMY copy number. Zoomed scatterplots for the four families with significant negative associations in Fig. S5 (Charlie, Tigger, MIR, and total DNA transposons). Axes, point colors, fit lines, and statistics are as in Fig. S5.

bioRxiv preprint doi: https://doi.org/10.1101/2025.08.14.670395; this version posted August 15, 2025. The copyright holder for this preprint (which was not certified by peer review) is the author/funder, who has granted bioRxiv a license to display the preprint in perpetuity. It is made available under aCC-BY 4.0 International license.

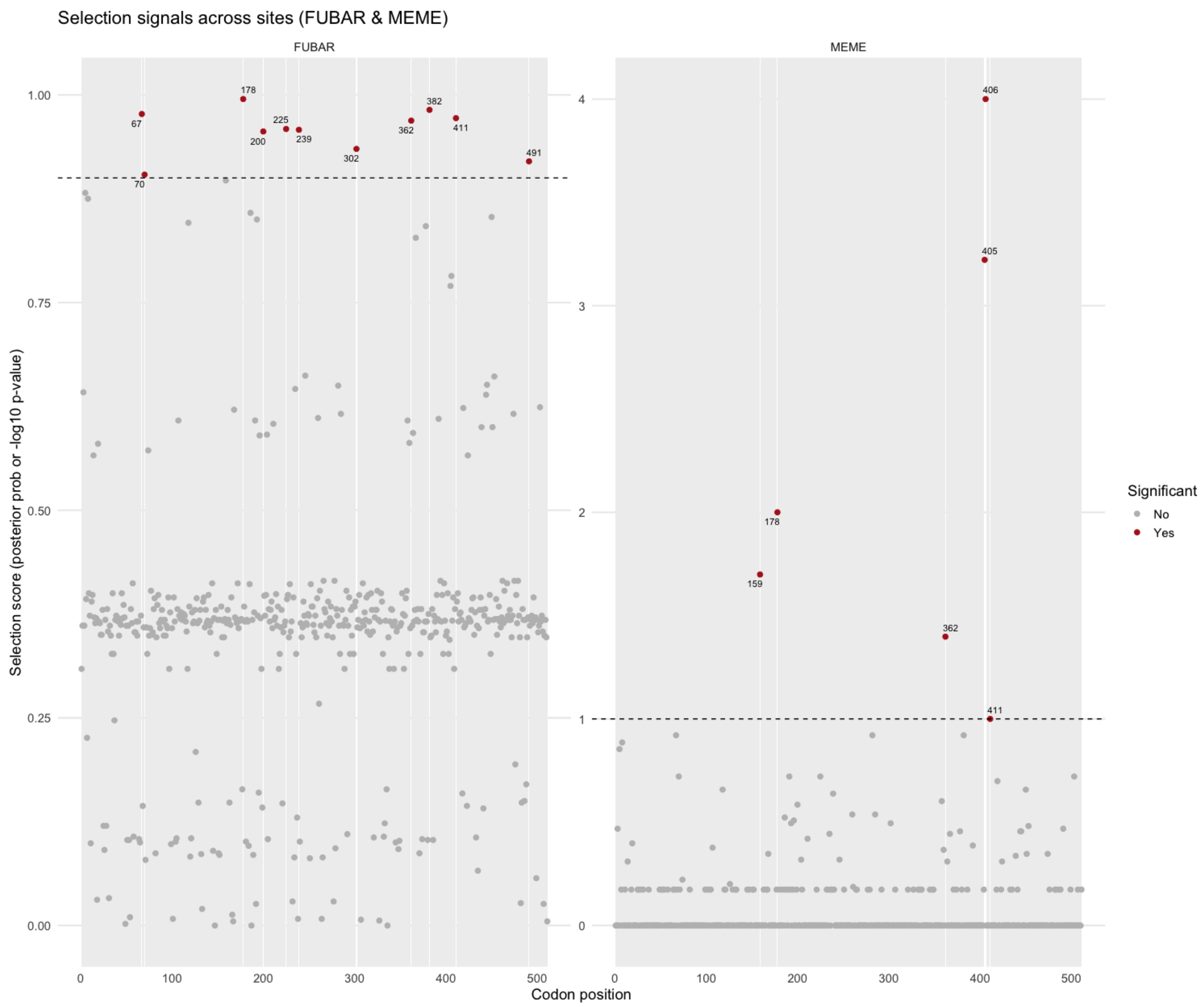

Fig. S8.

Site-level signals of selection across amylase coding sequences. Left: FUBAR posterior probability that ω>1 at each codon; the dashed line marks the 0.90 significance threshold. Right: MEME $-\log_{10}(P)$ for episodic selection; the dashed line marks P=0.10. Red dots denote sites called significant by each method (e.g. MEME: 159, 178, 362, 405, 406, 411; FUBAR: multiple sites ≥0.90). Analyses were run on the codon alignment of Old World monkey and great ape *AMY* paralogs.

bioRxiv preprint doi: https://doi.org/10.1101/2025.08.14.670395; this version posted August 15, 2025. The copyright holder for this preprint (which was not certified by peer review) is the author/funder, who has granted bioRxiv a license to display the preprint in perpetuity. It is made available under aCC-BY 4.0 International license.

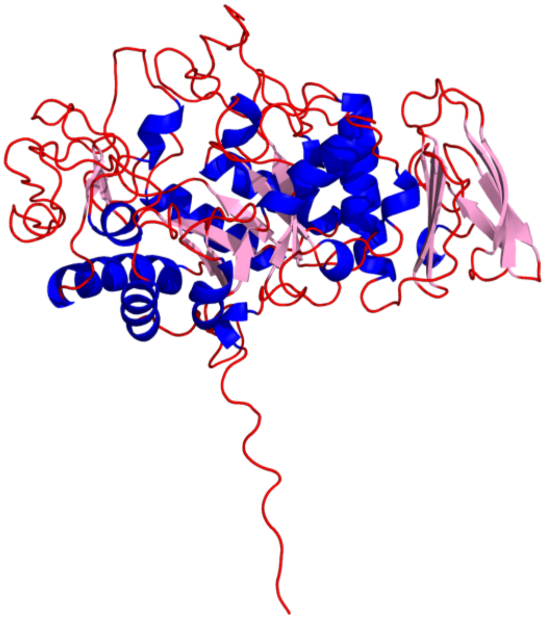

Fig. S9.
Predicted 3D structure of olive baboon *AMYp2*. AlphaFold2 model rendered as a PyMOL cartoon with α-helices in blue, β-strands in light pink, and loops/coil in red.

bioRxiv preprint doi: https://doi.org/10.1101/2025.08.14.670395; this version posted August 15, 2025. The copyright holder for this preprint (which was not certified by peer review) is the author/funder, who has granted bioRxiv a license to display the preprint in perpetuity. It is made available under aCC-BY 4.0 International license.

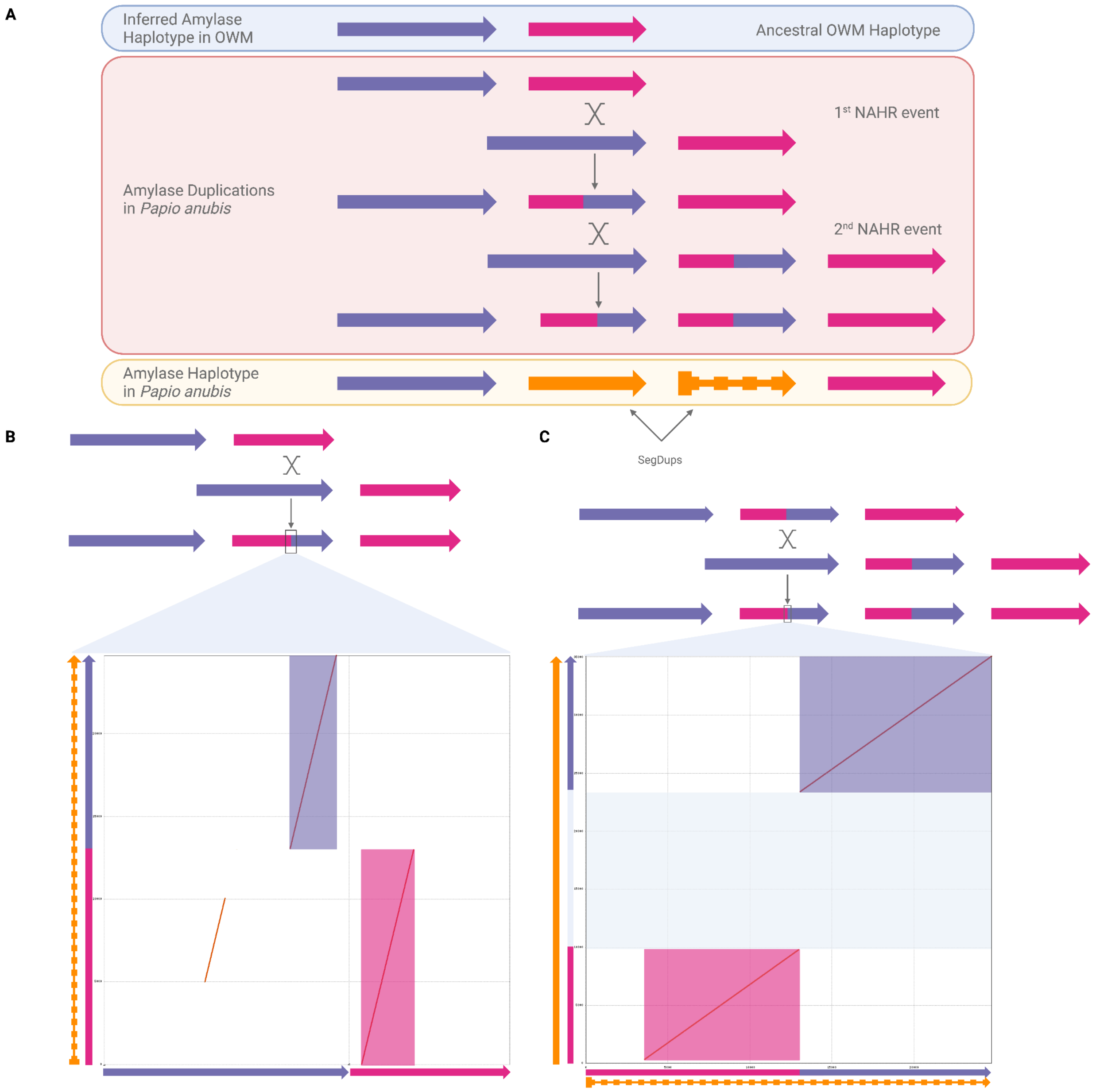

Fig. S10.

Figure S10. Stepwise NAHR duplications shaping the olive baboon amylase locus. The locus is partitioned into four duplicated segments, each drawn as a colored arrow to indicate block identity and orientation; colored arrows denote segments, not individual genes. Segment 1 (purple arrow) carries *AMY2B*, segment 4 (pink arrow) carries *AMY1'*, segment 3 (orange-dashed arrow) carries *AMYp1*, and segment 2 (orange arrow) harbors *AMYp2*. (A) Pairwise NUCmer alignments and dotplots show segment 3 is a mosaic of segment 1 and segment 4, consistent with a first NAHR event between segments 1 and 4 that created the *AMYp1*-containing block. (B) Segment 2 (which hardbours *AMYp2*) is near-identical to segment 3, except for an ~10 kb DNA stretch absent from segment 3. Because this interval is flanked by long N stretches in the olive baboon assembly, we cannot determine whether it reflects a true deletion or a scaffolding artifact.

bioRxiv preprint doi: https://doi.org/10.1101/2025.08.14.670395; this version posted August 15, 2025. The copyright holder for this preprint (which was not certified by peer review) is the author/funder, who has granted bioRxiv a license to display the preprint in perpetuity. It is made available under aCC-BY 4.0 International license.

These dotplots and cartoon schematic representation support a two-step NAHR model: NAHR between segments 1 and 4 produced segment 3 (harboring *AMYp1*), followed by NAHR between segment 1 and segment 3 produced segment 2 (harboring *AMYp2*), yielding the present-day order *AMY2B AMYp2*, *AMYp1*, *AMY1′* in olive baboon. Segments were defined from self-alignments/BISER and compared with NUCmer dotplots as described in Methods.

bioRxiv preprint doi: https://doi.org/10.1101/2025.08.14.670395; this version posted August 15, 2025. The copyright holder for this preprint (which was not certified by peer review) is the author/funder, who has granted bioRxiv a license to display the preprint in perpetuity. It is made available under aCC-BY 4.0 International license.

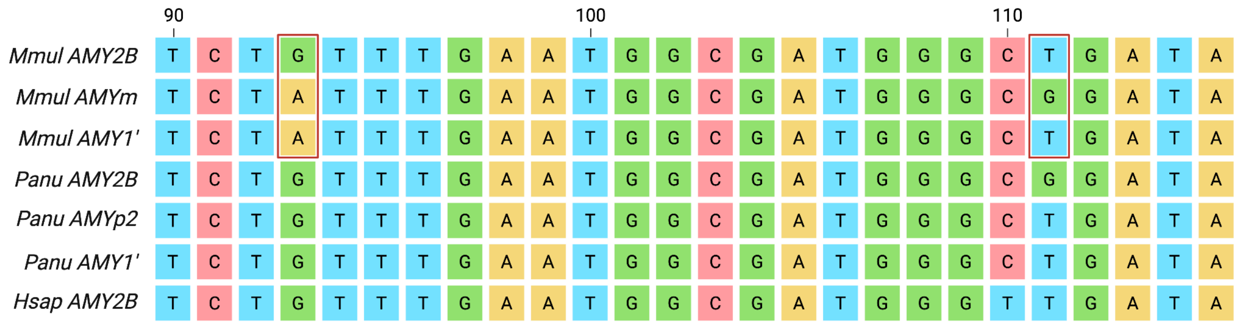

Fig. S11.

Example of the diagnostic polymorphisms used for paralog-specific expression in macaque and baboon. Alignment snapshot (codons ~90-10) across AMY paralogs shows fixed nucleotide differences used to assign RNA-seq reads to specific copies. Red outline boxes highlight parsimony informative sites in macaque (e.g. positions 93 and 111 distinguishing *AMY2B*, *AMYm*, and *AMY1'*). These markers underlie the SNP-aware quantification described in Methods.

bioRxiv preprint doi: https://doi.org/10.1101/2025.08.14.670395; this version posted August 15, 2025. The copyright holder for this preprint (which was not certified by peer review) is the author/funder, who has granted bioRxiv a license to display the preprint in perpetuity. It is made available under aCC-BY 4.0 International license.

Table S1.

Primate genome assemblies surveyed.
Columns: Assembly Accession (NCBI GCA/GCF), Assembly Name (submitter label), Organism Name (NCBI taxon), Annotation Name (if/when available), Assembly Stats Total Sequence Length (bp), Assembly Level (e.g. Scaffold/Chromosome), Assembly Release Date, WGS Project Accession, Assembly BioProject Accession. This table lists all assemblies screened; inclusion does not imply that the amylase locus was recovered on a single contiguous contig.

Table S2.
Species represented by multiple genome assemblies (n=16 species; 2-4 assemblies per species).
For each assembly of these species, we report: Assembly Accession, Assembly Name, Organism Name, Annotation Name (if available), Total Sequence Length, Assembly Level, Assembly Release Date, WGS Project Accession and BioProject Accession. This table is descriptive and does not include locus contiguity or copy-number estimations.

Table S3.
Genome assemblies with a contiguous amylase locus (n=69).
Assemblies listed here are the subset in which the amylase interval (RNPC3 5′ flank to AMY1C 3′ flank) was recovered on a single, gap-free contig/scaffold and used for locus-level analyses (synteny, dotplots, and copy-number reconstruction in Fig. 1A). For each assembly we report Assembly Accession, Assembly Name, Organism Name, Annotation Name (if available), Total Sequence Length, Assembly Level, Assembly Release Date, WGS Project Accession, BioProject Accession, and N50. The N50 value is the assembly-wide contig/scaffold N50 (bp) as reported by NCBI at the time of download.

Table S4.
Representative assemblies used for downstream analyses (one per species; 53 species).
From the 69 genomes with a contiguous RNPC3-AMY1C interval (Table S3), we selected one assembly per species for all downstream analyses (e.g. synteny analysis, dotplots, breakpoint mapping, TE quantification within the locus and species-level summaries).

Table S5.
Transposable-element (TE) composition at the amylase locus versus genome-wide across 53 primate assemblies.
For each species, genome-wide TE content is compared to TE content within the curated amylase locus (RNPC3 5′ flank to AMY1C 3′ flank; gap-free contigs only). Annotations were generated with RepeatMasker (v4.1.5; -species primates). Percentages are the fraction of bases masked ×100; ratios are unitless enrichments calculated as (% masked in amylase locus) / (% masked genome-wide), where values >1 indicate enrichment and <1 indicate depletion at the locus. The copy number column provides the number of AMY paralogs in that assembly's locus.
Columns: Species - scientific name; General Class - phylogenetic clade (e.g., OWM, NWM, tarsier, ape); copy number - AMY paralog count; % masked (Genome wide / Amylase locus / Ratio) - total TE burden; then, for each TE group/family, triplets of genome-wide %, amylase-locus %, and Ratio are reported: SINEs (overall), Alu, MIR; LINEs (overall), LINE1, LINE2, L3/CR1; LTR elements (overall), ERVL, ERVL-MaLRs, ERV class I, ERV class II; DNA elements (overall), hAT-Charlie, TcMar-Tigger.

bioRxiv preprint doi: https://doi.org/10.1101/2025.08.14.670395; this version posted August 15, 2025. The copyright holder for this preprint (which was not certified by peer review) is the author/funder, who has granted bioRxiv a license to display the preprint in perpetuity. It is made available under aCC-BY 4.0 International license.

Table S6.
Predicted functional impact of amino-acid substitutions in primate amylase paralogs.
SNVs are listed with GRCh38 coordinates (CHROM, POS, REF, ALT), the mapped UniProt protein (uniprot_id), and the study paralog (transcript_id). protein_variant shows the amino-acid change (RefAA-position-AltAA; e.g. K2Q). am_pathogenicity is a 0-1 predicted impact score (higher: larger effect), and am_class is its category (e.g. likely_benign, ambiguous, likely_pathogenic etc.) Only substitutions unique relative to the UniProt sequence are included.

Table S7.
RNA-seq sample metadata for olive baboon and rhesus macaque tissues.
Each row lists the specimen and tissue used for transcriptomics: SampleID, Species (olive baboon or rhesus macaque), Tissue (parotid, submandibular, sublingual, pancreas, liver), preservation Method (e.g. FlashFrozen), body side (Left/Right) when paired tissues were sampled, Age (years) and Sex. Samples from five olive baboons and six rhesus macaques underpin all expression analyses reported in the manuscript.

Table S8.
Predicted transcription factor binding sites (TFBS) in amylase promoters.
TFBS identified by FIMO (MEME Suite v5.5.8) using the JASPAR 2024 CORE non-redundant vertebrate library. Promoters were scanned as 170-bp windows (100 bp upstream to 70 bp downstream of the TSS). Only matches with $P < 1\times10^{-4}$ are reported (q-values shown for FDR control). Columns: motif_id (JASPAR matrix ID), motif_alt_id (TF symbol), sequence_name (promoter window: gene and species), start/stop (1-based coordinates within the promoter window), strand (motif orientation), score (FIMO match score), p-value (match probability), q-value (Benjamini-Hochberg adjusted p-value), matched_sequence (genomic k-mer). The full table contains 262 sites (108 unique motifs) across promoters of nine amylase paralogs in human, rhesus macaque and olive baboon.

Table S9.
Segmental-duplication calls in the olive baboon amylase locus (BISER v1.4).

Table S10.
Segmental-duplication calls in the rhesus macaque amylase locus (BISER v1.4).

Table S11.
aBSREL branch‑level tests of positive selection.
HyPhy v2.5.48 aBSREL results on AMY paralog codon alignments (olive baboon, rhesus macaque, great apes). P values are Holm-Bonferroni-corrected LRTs. Columns: branch (Species_Gene; NodeXX=internal), tested, p-value (evidence for $\omega>1$), sites (positively selected classes), rates (ω classes), ω distribution (class ω with proportions), ω plot (mean ω, CoV). Branches with $p<0.05$ indicate episodic diversifying selection, notably the internal OWM branch and *Papio anubis AMYp2* (Fig. 5B).

Table S12.
MEME (HyPhy) sites under episodic diversifying selection.
All tested sites are listed. Columns: Site (1-based codon), Partition (alignment partition), α

bioRxiv preprint doi: https://doi.org/10.1101/2025.08.14.670395; this version posted August 15, 2025. The copyright holder for this preprint (which was not certified by peer review) is the author/funder, who has granted bioRxiv a license to display the preprint in perpetuity. It is made available under aCC-BY 4.0 International license.

(synonymous rate), β−/p− (background dN and its weight), β+/p+ (episodic dN>1 and its weight), LRT (likelihood-ratio statistic), p-value (BH-FDR), # branches under selection (branches inferred with ω>1 at this site), Total branch length (from HyPhy), MEME LogL and FEL LogL (site log-likelihoods), Variation p (within-site rate-variation test). Sites with $p < 0.10$ indicate episodic selection.

Table S13.
FUBAR site-wise tests for pervasive selection.
Columns: Site (1-based codon), Partition (alignment block), α (dS), β (dN), β−α (effect size), Prob[α>β] (purifying selection), Prob[α<β] (positive selection), BayesFactor[α<β] (support for positive selection). Posterior summaries are from the FUBAR run described in Methods. Sites with Prob[α<β] ≥ 0.90 are called as positively selected; Prob[α>β] ≥ 0.90 indicates purifying selection.

Table S14. RELAX branch-wise relaxed selection test.
Columns: Branch name (terminal/internal label), Branch partition (Reference=background; Test=foreground), Branch length (expected substitutions/site), k (selection-intensity parameter; k>1 = intensified selection, k<1 = relaxed, k≈1 = no change). The designated foreground (Test) branch is *Papio anubis AMYp2*; model fitting and LRT significance are described in Methods. Test for selection intensification (K = 2.63) was significant (p = 0.000, LR = 15.27).
